# The Dammiss EEG Score: A New System to Quantify EEG Abnormalities and Predict the Outcome in Asphyxiated Newborns

**DOI:** 10.3390/jcm14061920

**Published:** 2025-03-12

**Authors:** Fabrizio Ferrari, Carolina Bondi, Licia Lugli, Luca Bedetti, Isotta Guidotti, Federico Banchelli, Laura Lucaccioni, Alberto Berardi

**Affiliations:** 1 Neonatal Intensive Care Unit, Department of Medical and Surgical Sciences for Mothers, Children and Adults, Modena University Hospital, 41125 Modena, Italy; ferrarifabrizio36@gmail.com (F.F.); lugli.licia@aou.mo.it (L.L.); bedetti.luca@aou.mo.it (L.B.); guidotti.isotta@aou.mo.it (I.G.); alberto.berardi@unimore.it (A.B.); 2 Post-Graduate School of Paediatrics, Department of Medical and Surgical Sciences for Mothers, Children and Adults, Modena University Hospital, 41125 Modena, Italy; 3 Unit of Statistical and Methodological Support to Clinical Research, University Hospital of Modena, 41125 Modena, Italy; federico.banchelli@gmail.com; 4 Unit of Paediatrics, Department of Medical and Surgical Sciences for Mothers, Children and Adults, Modena University Hospital, 41125 Modena, Italy; laura.lucaccioni@unimore.it

**Keywords:** EEG monitoring, perinatal asphyxia, hypoxic–ischemic encephalopathy (HIE), therapeutic hypothermia (TH), EEG-polygraph, neuro developmental outcome, full term newborn

## Abstract

**Background:** The aim of the study was to evaluate a novel EEG scoring system as a diagnostic and prognostic tool for brain injury in infants who had experienced perinatal asphyxia. **Methods:** The scoring system, based on a semi-quantitative approach, encompassed seven EEG parameters and their aggregate Dammiss score (DS) measured across seven time points (6 h, 12 h, 24 h, 48 h, 72 h, 78 h, and 2 weeks). The EEGs of 61 full-term newborns affected by perinatal asphyxia and treated with therapeutic hypothermia were evaluated. **Results:** The EEG parameters were correlated with the outcome at 2 years of age: 41 infants showed normal development; 16 presented with mild neurological abnormalities; and 4 developed cerebral palsy. Key EEG features—such as maturational patterns, sleep states, interburst interval, burst morphology and DS at 6 h of life—were highly predictive of outcomes. Correlations were also observed for sleep states, burst morphology, and DS at 12 and 24 h. Notably, burst amplitude and seizure did not correlate with outcome. Additionally, EEG recovery—observed in all patients—was temporarily impaired by seizures in 18% of the cooled infants. **Conclusions:** The EEG findings within the first 6 h of life were the most predictive of neurodevelopmental outcomes. The DS and EEG maturational features emerged as the most robust indicators of prognosis.

## 1. Introduction

Despite significant improvements in perinatal care, perinatal asphyxia (PA) remains a leading cause of death and severe neurodevelopmental issues in full-term newborns. PA causes hypoxic–ischemic encephalopathy (HIE) in about 1.6/1000 live newborn infants. Following resuscitation, it is crucial to assess whether the newborn infant has experienced a hypoxic–ischemic injury. If confirmed, neuroprotective therapeutic hypothermia (TH) must be initiated within six hours of birth [1].

To identify infants in need of TH, comprehensive neurologic evaluation and neurophysiological assessments are essential. However, interpreting neurological examinations in the first few hours post-birth can be challenging, as distinguishing between mild and moderate neonatal encephalopathy is often difficult due to rapidly changing neurological symptoms. Additionally, in some cases, neurological manifestations of brain injury may emerge several hours after birth [2,3].

Most neonatal units employ amplitude-integrated electroencephalography (aEEG) to determine which infants require TH. Abnormal aEEG patterns are classified into four categories: discontinuous normal voltage, burst suppression, continuous low voltage, and flat trace [4,5,6,7]. However, this approach has several limitations, including interference from muscular artifacts caused by shivering and tremors associated with TH [4]. Moreover, a minimum duration of 30 to 50 min is necessary for aEEG abnormalities to manifest. Brief, focal, and low-voltage seizures can be missed by aEEG. Conventional EEG recordings—typically performed as polygraphic recordings—along with video-EEG recordings demonstrate greater efficacy in differentiating tremors and shivers from true abnormal EEG features and identifying seizures, particularly focal, short, and subclinical ones [8,9]. Conventional EEG also effectively recognizes background patterns, maturational features, various sleep states, and interburst intervals, as well as the burst morphology and duration. As a result, conventional video-EEG is regarded as the gold standard for the long-lasting neurophysiologic monitoring of infants with HIE before and during TH [10]. This method can reveal the severity of electrophysiologic abnormalities immediately after the brain insult. Experimental studies indicate that EEG can document abnormal electrical activity following an acute brain injury within 10 to 20 min [11]. Documenting EEG changes in the hours that follow is also critical for assessing the severity of neonatal encephalopathy. While many studies suggest EEG can predict outcomes in severe HIE, its effectiveness in mild to moderate encephalopathy cases remains questionable. Most of these investigations [12,13,14] analyzed only a limited cohort of HIE patients treated with cooling and utilized various EEG scoring systems, leaving ambiguity about which system most effectively predicts outcomes.

Our work involved a careful study and review of validated EEG classifications on populations of term infants with HIE currently in use. From the study of such classification schemes, we thus sensed a major gap present in neonatal neurophysiology: this is an area where a consensus is urgently needed [5,7]. The purpose of this article is to propose a single classification system for the EEGs of infants with HIE; we produced it based on the study of existing schemes and optimized through our insights and experience in the field. Once we identified the seven quantifiable parameters that were most significant to us, we tested them and applied them to the population in question.

For this study, we reviewed EEG recordings of all infants diagnosed with HIE who underwent TH from 2009 to 2018. We developed a new classification system based on the assessment and semi-quantification of seven EEG parameters identified in the literature as potential indicators of brain injury. The abnormalities concerning these seven EEG parameters and their overarching score (termed the global Dammiss score, “DS”) were correlated with outcomes at 24 months of age [15]. Based on the Prechtl optimality score [16] and the relevant literature, we selected seven EEG features for this classification: burst duration, burst amplitude, burst morphology, maturational features, interburst interval (IBI) duration, sleep states, and electrical seizures. The optimality score enables a semi-quantitative assessment of the EEG quality; a higher score reflects optimal performance and generally correlates with better EEG quality. The present study seeks to address three key questions:

1. At which time point (6 h, 12 h, 24 h, 48 h, 72 h, 78 h and 2 weeks) do the EEG parameters best correlate with HIE outcomes?
2. Among the seven EEG features, which demonstrates the highest accuracy in predicting the neurodevelopmental outcomes of HIE infants 24 months after birth?
3. What additional insights can continuous EEG monitoring provide?

## 2. Methods

We conducted a monocentric retrospective study and reviewed the clinical findings of 61 full-term infants born in or referred to our tertiary-level neonatal intensive care unit (Modena University Hospital NICU) from 31 January 2009 to 31 August 2018 who were affected by HIE grade II or III according to modified Sarnat and Sarnat classification [17] and were treated with TH because of perinatal asphyxia. Three selection criteria were derived from the Italian Recommendations on assistance to newborns with hypoxic–ischemic encephalopathy as candidates for hypothermic treatment [18]. The first criterion was intrapartum hypoxia, which was defined by the existence of at least one of the following conditions: (a) APGAR score ≤ 5 at 10 min of life, (b) need to continue ventilation with a mask or endotracheal tube for at least 10 min, and (c) severe acidosis within 60 min of birth, defined as a cord or any arterial/venous pH ≤ 7, or a base deficit ≥ 12 mmol/L. The second criterion was the presence of moderate to severe encephalopathy evaluated within the first two hours of life [19]. The third and last criterion was the presence of moderate to severe abnormalities detected on early conventional EEG using the EEG classification adapted from Murray et al. [6]. Congenital malformations, chromosomal abnormalities, connatural infections or infections of the central nervous system, metabolic disorders, cerebral malformations, postnatal collapse, neonatal stroke, and diagnosed or suspected epileptic encephalopathies were considered as exclusion criteria. A control group was not assigned in this study.

TH was performed by cooling patients to a rectal temperature of 33.5 °C for 72 h using CritiCool™ (MTRE, Charter Kontron, Milton Keynes, UK), followed by slow rewarming (+0.5 °C/h) from 72 to 78 h. TH was used from 1 March 2009 onwards, and no additional neuroprotective therapies (such as stem cell therapy, melatonin, or xenon) were administered during the study period. Table 1 shows the obstetric pre- and perinatal data and demographic data of the final study population. During TH, all 61 infants were sedated with morphine (boluses of 50–100 y/kg every 4–6 h) or fentanyl (continuous infusion of 0.5 y/kg/h).

EEG monitoring was initiated within the 2nd and 6th hour of life and continuous monitoring was performed up to 84 h after birth. This was repeated in the second week of life (from the 8th to the 10th day). The scores at single time points were assessed at 6, 12, 24, 48, 72, and 78 h after the beginning of the EEG by counting the dominant pattern spanning 30 min. The last EEG was performed during the second week of life. All infants were followed up at the Neonatal Unit of our facility, according to the neuropsychological follow-up protocol at 3, 6, 12, and 24 months of life.

The follow-up assessments were performed by four experienced neonatologists trained in developmental neurology, a developmental psychologist, and a physical therapist. All major postural and motor steps were video recorded and scored in real time or after reviewing the videos using the standard neurological examination by Amiel Tison and Grenier [20] and an extension of Touwen’s criteria [21]. Griffiths Mental Developmental Scales (GMDS-R) were also administered. GMDS-R (0–2 years) provides a global development quotient (DQ) of infants’ abilities with a mean of 100.5, a standard deviation (SD) of 11.8, and five subscale quotients (locomotor, eye and hand coordination, personal and social, hearing and language, and cognitive performance). In case of any disagreement regarding the neurologic items scored at 24 months, the videos were reviewed by the members of the follow-up team and a consensus was reached after discussion. The neurologic outcome was defined as “normal” when no neurological signs were observed, and the developmental quotient (DQ) was higher than 85. It was defined as “mild sequelae” if the child showed mild motor impairment [walked independently but was clumsy and/with poor balance without evidence of cerebral palsy (CP), or mild mental impairment (DQ between 70 and 85) or hearing impairment with no need for a amplifier, or a clear speech delay. The third outcome considered was CP, which was defined as a permanent but not unchanging disorder of movement or posture or both and of motor function, further classified as spastic (diplegia, hemiplegia, or quadriplegia), dystonic, or athetoid. In case of CP, the severity of the motor impairment was scored according to the Gross Motor Function Classification System (GMFCS) [22].

### 2.1. EEG Criteria

We selected seven EEG parameters after referring to the available literature [6,13,21,22,23,24]: burst duration, burst amplitude, burst morphology, maturational features (frontal sharp transient or slow anterior dysrhythmia), IBI duration, sleep states, and seizures. The definitions of the seven EEG parameters are provided in the Appendix A.

The sum of the scores of seven individual EEG parameters was considered as the eighth parameter. The term “Dammiss” is an acronym derived from the initials of each of the seven EEG parameters: “D” for duration of the burst, “A” for amplitude of the burst, “M” for burst morphology, “M” for maturational features, “I” for inter-burst interval, “S” for sleep states, and “S” for seizures.

To proceed with the evaluation of the single EEG trace, it is necessary to assign a score from 0 to 2 for each parameter (where 0 corresponds to severe abnormalities, 1 to mild abnormalities, and 2 to normal EEG trace).

**Figure A1:**
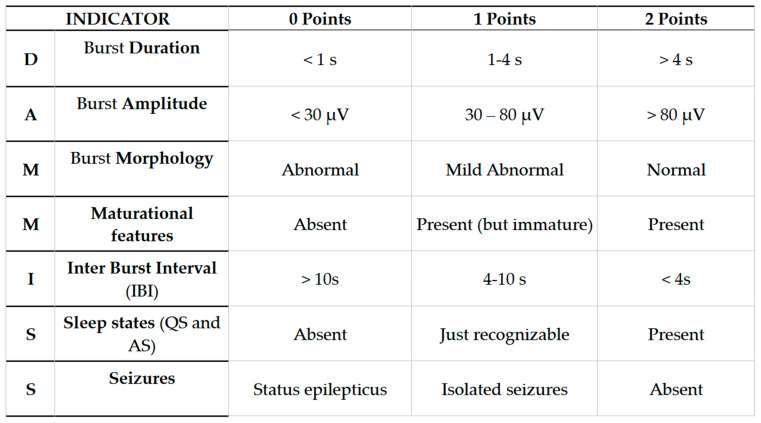
Dammiss Score.

Once the values have been assigned—similarly to other scores, such as the more famous Apgar Score—it is possible to obtain an overall value given by the sum of the individual parameters. The more the trace appears poor and altered, the lower the overall score obtained; vice versa, the more the trace is organized and free of anomalies, the higher the overall score. This value ranges from 0 to 14 points, where 0 corresponds to a severely pathological trace and 14 represents a well-organized EEG trace. For more details, see Figure A1 and Appendix A below.

### 2.2. Statistical Analysis

The numerical variables were described as the mean and standard deviation (SD), whereas the categorical variables were presented as the absolute numbers and percentages. Correlations between variables were assessed by using Spearman’s rank correlation coefficient (Scc), which ranges from −1 (maximum negative correlation) to 0 (no correlation) and 1 (maximum positive correlation). Predictive accuracy was assessed with the receiver operating characteristic (ROC) curve analysis and measured as the area under the curve (AUC). AUC values range from 50% (casual accuracy) to 100% (perfect accuracy). All analyses were performed with R 3.6.3 statistical software (The R Foundation for Statistical Computing, Vienna, Austria) and the statistical significance was set at *p* < 0.05.

### 2.3. Study Group

According to the inclusion criteria, 70 full-term infants were enrolled initially, but 9 of them were subsequently excluded. Among these 9 infants, 5 were excluded because of missing EEG data, 2 suffered from early collapse, and 2 infants did not undergo the follow-up check at 24 months. The majority of the study population were Caucasian (77.0%) and male (59%). The perinatal data of the study group are reported in Table 1. Among 61 enrolled newborns, 22 experienced severe HIE, 35 experienced moderate HIE, and 4 experienced mild HIE. As for the outcome at 24 months, 41 infants showed a normal motor and development quotient, 16 showed mild neurological abnormalities (predominantly clumsiness and speech delay), and 4 had cerebral palsy. Severity of HIE, total DS, and the neurological outcome at 24 months evaluated with the neurological assessment and the neurodevelopmental quotient (Griffiths scale) are reported in Table 2.

## 3. Results

In order to answer our first research question, we investigated the time point at which the EEG best correlated with the outcome. The Spearman rank (R) assessment between the seven single EEG components, in addition to the global score at the seven time points (6 h, 12 h, 24 h, 48 h, 72 h, 78 h, and second week) and the outcome, is shown in Table 3. The highest correlation coefficient was found at 6 h for all the EEG features except for burst amplitude and seizures, which never correlated with outcome at any time point. Rank ordering of Spearman’s coefficient (R) from the highest to the lowest value at the single time points revealed that the global DS and burst morphology (R 0.45) had the highest correlation, followed by sleep state (R 0.42), maturational features (R 0.36), burst duration (R 0.29), and interburst interval (R 0.28).

**Figure 1:**
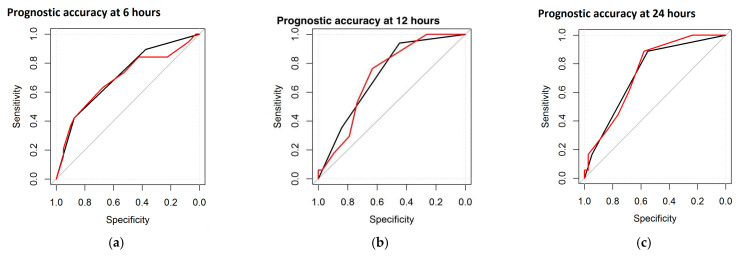
ROC curves at different time points: at 6 h (**a**), at 12 h (**b**), and at 24 h (**c**). Black line refers to maturational features and the red line refers to the DS.

As for the entity and the duration of the correlation, Spearman’s coefficient was highest at 6 h, decreased by 12 and 24 h, and remained significant at these three epochs for three parameters, including sleep states, burst morphology, and global DS. Furthermore, it became positive again at 72 and 78 h for two parameters (sleep states and global DS). The IBI duration showed positive correlations at 6 and 78 h. Maturational features and burst duration were positive only once, at 6 h. A second statistical computation, the ROC curve, was performed to test the prognostic accuracy of the single EEG features and the DS with respect to the neurological outcome. The neurological outcome was divided into Normal and Abnormal (including severe and mild abnormalities). Within the study group (n = 61), the highest accuracy was found for maturational features and global DS, reaching 73.5% and 74.6% AUC at 24 h, respectively. Figure 1 represents the ROC curve, with the black line referring to the maturational features and the red line referring to the total DS (at 6, 12, and 24 h).

The ROC curves revealed the best prognostic accuracy of the global DS and maturational features. The percentages of prognostic accuracy of the global DS and maturational features are reported in Table 4.

Regarding the third research question that we attempted to answer, a progressive improvement of the EEG recovery curve was documented (see Table 2). The entity of the score changed during the TH window for all the EEG parameters, with a clear trend of increase from 6 to 78 h and up to the second week. Moreover, some infants showed a transient arrest/deterioration of their EEG recovery, which lasted for one or two time periods at most and consisted of a global DS that was one to two points lower compared to the previous time points (see Table 2). Patient numbers 1, 25, 45, and 61 are good examples of this phenomenon. This severe drop either consisted of a progressive deterioration (case 31 and case 25) or a sudden deterioration (case 3 at 24 h, case 50 at 78 h, and case 54 at 12 h). When the cause of this deterioration in the EEG recovery curve was investigated, the onset was found to be predominantly coincident or following electrical or electroclinical seizures in nine of the thirteen infants. For example, in case 50, the background EEG suddenly deteriorated during the rewarming phase, with the DS jumping from 11 at 72 h to 6 at 78 h, and the EEG revealed electrical discharges that were interrupted by the restarting of cooling and by the administration of an antiepileptic drug (iv phenytoin). In case 54, the DS jumped from 12 at 6 h to 5 at 12 h and the EEG revealed the emergence of electrical subclinical discharges.

## 4. Discussion

This study introduces the Dammiss score as an innovative semi-quantitative method for assessing EEG abnormalities and predicting outcomes in asphyxiated cooled infants. The purpose of this work is to introduce a standardized and easily reproducible EEG scoring system. Multiple scores are currently in use and there is still no clear consensus on them. In a future where artificial intelligence may also play a role in EEG trace evaluation, the use of clear and reproducible criteria is essential. The DS requires short training and is easy to use even by inexperienced operators, precisely “for dummies”, hence the name “Dammiss score”. Our findings indicate that certain EEG parameters at six hours of life—specifically sleep states, IBI, burst morphology, and DS—are strongly correlated with clinical outcomes. This time point appears to be the most critical for evaluating brain damage severity. Importantly, sleep states and burst morphology also retained predictive value at 12 and 24 h, underscoring that the first day, particularly the initial six hours, serves as the best indicator of brain injury severity.

In circumstances where continuous EEG monitoring during the TH window is impractical, we recommend performing at least two episodic EEGs: one within the first six hours and another at 12 or 24 h. These targeted assessments with cEEG, combined with aEEG traces performed for the rest of monitoring, can yield prognostic insights comparable to those obtained through continuous monitoring with cEEG. The EEG during the first six hours is vital, not only to determine candidates for TH but also to distinguish the HIE severity and to identify infants who require intensive monitoring and management from 12 to 24 h, particularly due to the higher incidence of neonatal seizures during this timeframe.

Contrary to previous studies [24,25,26,27] that focus on burst amplitude as a major marker of brain damage, our research presents a new perspective. Notably, burst amplitude and seizures showed no correlation at any of the seven assessed time points. The expected correlation between seizures and poor outcomes in neonates has changed in the context of TH, which has been shown to reduce both seizure frequency and severity [15,28]. Interestingly, in our research, we observed significant declines in DS in infants experiencing electrical discharges, with some cases showing a transition to inactive or severely discontinuous EEG patterns lasting a few hours. This suggests that acute EEG change necessitates a thorough review of prior EEG activity to identify potential unnoticed seizures, especially if they appear brief and focal [29]. The most compelling predictors of neurodevelopmental outcomes were found to be the DS and maturational EEG features at six hours of life.

The presence of maturational features (frontal sharp transients and anterior slow dysrhythmias), traditionally viewed as normal features if associated with normal EEG activity background, has now been identified as an indicator of positive outcomes, even when they appear early in time, immature and coupled with abnormal EEG.

Finally, our investigation into the eight EEG parameters of the DS suggests that their collective assessment over time enhances understanding of early anoxic–ischemic injuries and provides insights into the trajectory of EEG recovery. The diversity in EEG recovery patterns and the relationship between specific features and overall prognosis highlight the importance of these parameters in informing clinical decision-making and outcomes in neonatal care. Further research with larger cohorts is essential to explore these findings and their implications for managing HIE.

### Strengths and Limitations of the Study

The Dammiss score represents a novel tool to quantify EEG abnormalities and predict the outcome in asphyxiated newborns. The DS is innovative compared to the previous ones because it does not use a gestalt approach but adopts a new semi-quantitative approach based on definite parameters. This scoring system is not only straightforward for researchers to adopt, but it also offers critical advantages in the real-time monitoring and quantification of EEG features. However, our study has several limitations: first, the study population is small and the DS needs to be tested on larger populations to confirm its validity; second, we did not evaluate the possible effect of analgesic drug (opioid) administered during TH on the EEG; and third, in this study, EEG abnormalities were not evaluated in correlation with neuroimaging.

During the statistical analysis, we tried to identify a score cut-off corresponding to the risk stratification of having poor prognosis at 2 years of life. We divided patients into two groups: DS ≤ 8 and DS > 8. We conducted analyses of specificity, sensitivity, odds ratio, relative risk, and positive and negative predictive value for both groups, without reaching statistical significance given the small population (expressed as *p*-value < 0.05). Further studies that expand the population are needed, so that it may be possible to provide, as with other scores, a score cut-off that may be predictive of poor prognosis.

## 5. Conclusions

This novel EEG scoring system, grounded in seven parameters, reveals a strong correlation between maturational features, sleep states, burst morphology and duration, IBI, and DS during the first six hours of postnatal life with neurodevelopmental outcomes. Notably, burst amplitude and seizures showed no correlation at any of the seven assessed time points. The most compelling predictors of neurodevelopmental outcomes were the DS and maturational EEG features at six hours of life. Moreover, EEG recovery exhibited a non-linear progression, characterized by fluctuations, temporary plateaus, or prolonged regressions, frequently triggered by electrical seizures.

## Figures and Tables

**Table 1:** Perinatal data (absolute numbers and percentage) of the study population.

	Perinatal Data
36 (59%)	Male, n (%)
	Auxological data at birth:
61.67 (27.07)	Mean percentile birth weight (SD)
66.32 (28.08)	Mean percentile birth length (SD)
39.75 (1.18)	Mean Gestational Age, weeks (SD)
	Mode of Delivery
21 (34.42%)	Vaginal delivery, n (%)
40 (65.5%)	Operative delivery, n (%)
15 (24.59%)	Vacuum, n (%)
3 (4.92%)	Kristeller, n (%)
3 (4.92%)	Kristeller + Sucker, n (%)
19 (31.15%)	Emergency Cesarean Section, n (%)
	Amniotic Fluid
20 (37.8%)	Limpid, n (%)
30 (56.6%)	Meconium stained, n (%)
3 (5.6%)	Hematic, n (%)
1.72 (1.35)	Mean APGAR SCORE at 1st minute (SD)
4.1 (1.68)	Mean APGAR SCORE at 5th minute (SD)
5.6 (1.65)	Mean APGAR SCORE at 10th minute (SD)
	Umbilical Cord Acidosis
6.93 (0.16)	Mean pH (SD)
−17.07 (5.54)	Mean Base Excess (SD)

**Table 2:** HIE grade, DS at the 7 time points, developmental and neurological outcome at 24 months in 61 HIE cooled full-term infants. (CP = cerebral palsy) (NA: not assessable; NP: not present).

Griffith DQ	Neurological Outcome at 24 Months	HIE GRADE		Global Dammiss Score at the 7 Time Points						Patient No.
			>7	78	72	48	24	12	6	
108	Mild Abnormal: clumsy	3	X	X	4	8	3	9	10	1
96	Normal	3	X	X	X	8	5	7	4	2
NA	Normal	3	14	X	11	9	1	7	6	3
75	Mild Abnormal: clumsy speech delay	3	X	X	10	9	9	9	4	4
111	Normal	3	13	X	X	7	9	7	6	5
104	Normal	3	13	11	10	8	6	5	6	6
49	Abnormal: CP	3	13	11	11	X	6	7	6	7
117	Normal	2	14	11	11	10	X	10	10	8
110	Normal	2	X	12	11	10	9	9	9	9
89	Normal	2	X	12	12	X	9	10	9	10
NA	Normal	3	12	X	7	9	9	8	6	11
49	Abnormal: CP	3	12	8	6	9	6	X	6	12
93	Abnormal: CP	3	X	12	11	9	5	4	3	13
101	Mild Abnormal: clumsy	2	11	7	8	5	3	5	4	14
105	Normal	2	X	11	11	10	9	9	10	15
112	Normal	3	12	11	10	8	5	2	X	16
106	Mild Abnormal: clumsy	2	13	12	10	9	X	X	4	17
111	Normal	3	14	12	12	12	11	10	9	18
110	Normal	2	12	12	11	11	10	13	10	19
112	Normal	2	12	12	11	11	10	13	10	20
77	Mild Abnormal: mental delay	3	11	6	5	5	2	5	2	21
85	Mild Abnormal: speech delay	3	14	12	9	8	5	4	6	22
102	Normal	2	14	X	13	10	11	10	9	23
89	Mild Abnormal: clumsy	2	14	11	10	12	5	6	2	24
117	Normal	2	13	13	9	5	7	9	8	25
117	Normal	2	12	12	12	12	12	11	11	26
100	Normal	2	12	11	11	10	9	8	7	27
106	Normal	2	X	12	10	9	X	8	8	28
110	Normal	2	12	11	8	5	9	10	8	29
109	Mild Abnormal: eye movement deficit	2	13	11	11	10	8	9	8	30
112	Normal	2	6	4	4	5	5	7	10	31
106	Normal	2	11	10	6	6	7	6	6	32
114	Abnormal	2	10	9	8	8	7	6	7	33
NA	Mild Abnormal: mild hearing loss	2	10	10	11	8	7	7	7	34
111	Normal	2	14	11	11	10	X	X	7	35
105	Normal	3	13	11	11	8	6	4	4	36
NA	Normal	2	X	11	11	9	8	4	2	37
109	Normal	2	X	11	11	10	10	10	9	38
108	Normal	2	12	9	9	7	9	9	7	39
88	Normal	2	10	10	7	9	8	9	10	40
112	Normal	2	13	13	11	10	10	9	7	41
93	Normal	2	X	8	8	8	6	5	6	42
83	Mild Abnormal: clumsy	2	X	10	10	9	9	6	5	43
102	Normal	2	X	10	10	X	10	8	9	44
110	Abnormal: CP	2	13	10	10	7	8	8	11	45
104	Normal	3	11	10	10	10	9	5	2	46
NA	Normal	3	14	10	10	9	9	9	9	47
104	Normal	2	13	11	10	10	9	NA	11	48
86	Mild Abnormal: speech delay	3	11	8	6	4	0	1	2	49
110	Normal	3	13	6	11	10	7	8	6	50
105	Normal	2	14	9	10	9	9	11	6	51
NA	Normal	2	12	7	7	7	5	6	7	52
103	Normal	2	NP	10	9	9	10	7	7	53
108	Normal	3	NP	12	9	8	8	5	12	54
119	Mild Abnormal: clumsy	23	14	12	11	9	8	6	8	55
85	Mild Abnormal: clumsy	2	X	8	8	8	8	7	X	56
115	Normal	1–2	9	4	2	4	5	4	5	57
100	Normal	1–2	14	9	8	7	8	8	8	58
105	Mild Abnormal: clumsy	1–2	X	9	8	8	8	6	6	59
74	Normal	1–2	11	11	8	9	8	8	9	60
111	Mild Abnormal: clumsy	2	14	13	10	3	7	8	8	61

**Table 3:** Spearman’s correlations between the DS features (M, BM, BD, BA, SS, I, S, and DS) and the DQ according to the Griffith’s Scale.

Griffith Tot								
Spearman Rank (R) and *p*-Value		6 h	12 h	24 h	48 h	72 h	78 h	2 nd Week
M	R	0.36	0.08	0.14	−0.05	0.23	0.15	−0.27
	*p*-value	0.0087	0.5939	0.3329	0.7089	0.1035	0.3149	0.0952
SS	R	0.42	0.33	0.28	0.17	0.32	0.34	0.19
	*p*-value	0.0018	0.0210	0.0503	0.2353	0.0222	0.0171	0.2609
I	R	0.28	0.22	0.18	−0.12	0.14	0.28	0.08
	*p*-value	0.0410	0.1248	0.2055	0.4109	0.3165	0.0453	0.6188
BD	R	0.29	0.19	0.19	0.09	0.14	0.01	−0.27
	*p*-value	0.0380	0.1964	0.1755	0.5464	0.3394	0.9557	0.1050
BA	R	0.18	0.13	−0.08	−0.20	−0.18	0.01	0.00
	*p*-value	0.2060	0.3635	0.6035	0.1690	0.2109	0.9682	1.0000
BM	R	0.45	0.35	0.36	0.03	0.13	−0.01	0.00
	*p*-value	0.0007	0.0137	0.0092	0.8420	0.3760	0.9657	0.9766
S	R	NA	0.19	0.16	0.13	−0.20	−0.09	NA
	*p*-value	NA	0.1663	0.2396	0.3572	0.1446	0.5315	NA
DS	R	0.45	0.31	0.26	0.07	0.29	0.33	0.07
	*p*-value	0.0008	0.0320	0.0731	0.6241	0.0402	0.0205	0.6601

**Table 4:** Area under the ROC curve (AUC).

	AUC at 6 h	AUC at 12 h	AUC at 24 h
Global DS	78.6%	74.6%	67.6%
Maturational Features	75.1%	73.5%	70.9%

**Figure A2:**
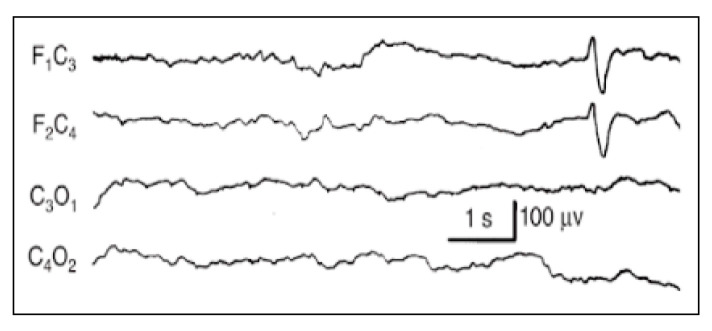
Frontal encoches.

**Figure A3:**
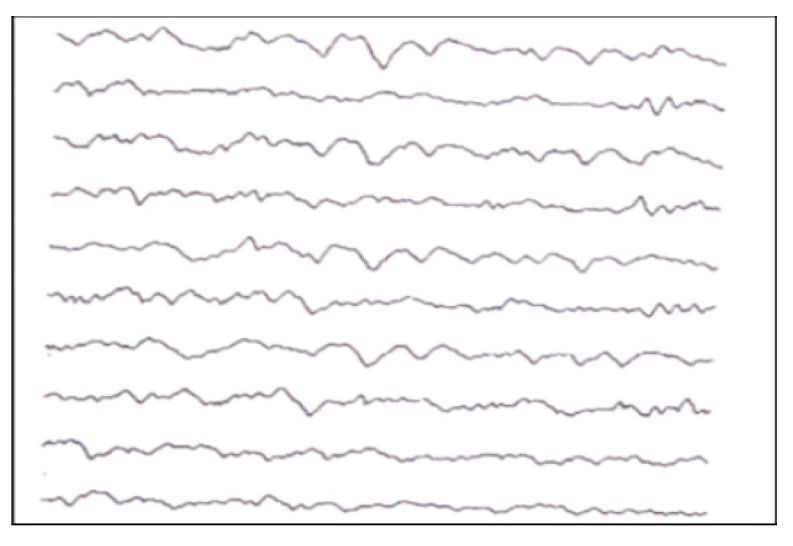
Anterior slow dysrhythmia.

**Figure A4:**
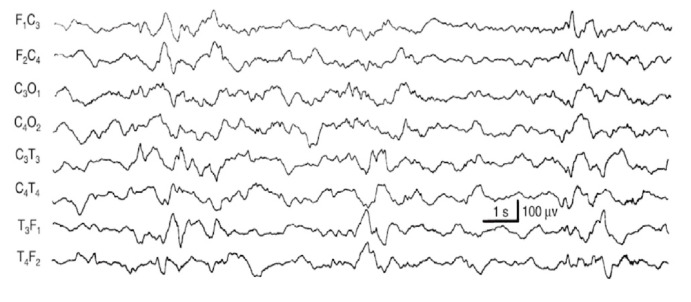
TA of a newborn.

## Data Availability

The original contributions presented in the study are included in the article, further inquiries can be to the corresponding author.

## Abbreviations

The following abbreviations are used in this manuscript: amplitude-integrated electroencephalography (aEEG), anti-epileptic drugs (AEDs), area under the curve (AUC), cerebral palsy (CP), Dammiss score (DS), developmental quotient (DQ), Gross Motor Function Classification System (GMFCS), hypoxic–ischemic encephalopathy (HIE), interburst interval (IBI), perinatal asphyxia (PA), receiver operating characteristic (ROC), Spearman’s rank, correlation coefficient (Scc), standard deviation (SD), therapeutic hypothermia (TH).

## Appendix A. Dammiss Score

The “Dammiss Score” is a new scoring system in which seven items are analyzed: burst duration, burst amplitude, burst morphology, maturational features (FSTs and ASD), IBI duration, sleep states (sleep–wake cycle), and seizures. The term consists of the acronym of the initial letters of the seven parameters evaluated. (Figure A1) [30,31,32].

- Burst Duration (BD): Expressed in seconds. We calculate the average considering three consecutive larger bouffées. The duration of the bouffées can be easily measured by evaluating the time on the abscissa of the recording (considered the bigger bouffées in the interval).
- Burst Amplitude (BA): Expressed in μV. The average is calculated on three consecutive larger bouffées. Through the EEG program used in our NICU, we measured the amplitude analyzing the peak–peak parameter (μV) on derivation C3–C4.
- Burst Morphology (BM): Essentially assessed by visual qualitative evaluation. Physiological bouffées are considered as those having three different physiological elements: sharp waves, slow delta waves (0.5–4 Hz), and slow theta waves (4–8 Hz). For undifferentiated bouffées or the absence of physiological features, a score of 0 was assigned.
- Maturational features (M): Given by visual qualitative assessment. We evaluated frontal encoches (or FSTs, frontal sharp transients) and ASD (anterior slow dysrhythmia). Frontal encoches or FSTs (Figure A2) are high-amplitude (250 μV) polyphasic waves in frontal regions with the tendency to spread to the posterior areas. They are generally symmetrical and synchronous in the two hemispheres. FSTs are physiological from 34 to 35 weeks. Anterior slow dysrhythmia (ASD): Delta waves (1–3 Hz) of 50–100 μV in short sequences lasting a few seconds, predominant in the frontal regions. (Figure A3). FSTs and ASD are typical of the first hours of life, but from the 10th day of life, the typical physiological patterns are TA, HVS, M, LVI, and Moyenne activity.

- IBI duration (I): Expressed in seconds. Since burst interval characterizes the discontinuous background, its duration (seconds) and aspects (physiological IBIs are not isoelectric or flat) can easily be evaluated. If different types of bouffées are present, consider the largest ones. At least three IBIs have to be present to consider the background as discontinuous. To assess duration, calculate the average. If IBIs are difficult to recognize, give a score of 1 point.
- Sleep States (SS): Evaluated by visual assessment. If EEG patterns agree with gestational age, behavioral states, and the sleep–wake cycle, they are signs of brain integrity, while the lack of this agreement can be considered a sign of brain dysfunction. The organization of sleep states is therefore another important window into the development and integrity of the CNS. In the newborn, the sleep cycle can be divided into five phases:
  1. Quiet sleep (QS) is mature at 36–37 weeks PMA. It can occur through two patterns: HVS (high-voltage slow), a medium-voltage pattern rich in delta activity, and TA (alternating pattern, “tracé alternant”) (Figure A4), which is a discontinuous pattern of 1–3 Hz and 50–100 μV, alternating with lower amplitude beta waves and theta activity bursts of 3–10 Hz lasting 3–5 s.
  2. Active sleep (AS) coincides with REM sleep. It consists of 2 patterns: M (mixed) and LVI (low-voltage irregular). M is a continuous activity which appears at the beginning of AS, characterized by theta and delta waves with some alpha waves, and voltage of 40–100 μV. When AS follows QS, it presents as LVI (20–50 μV), associated with beta/theta and alpha waves diffusely distributed.
  3. Quiet wakefulness
  4. Active wakefulness with general movements
  5. Crying
  In the second week of extra-uterine life, the QS is characterized mostly by HVS (high-voltage slow), which predominates over TA (tracé alternant). To analyze each sleep state, besides EEG patterns, we considered information resulting from breaths and eye movements measurement too. The AS is characterized by rapid eye movements (REM), body movements, continuous alterations of respiratory and cardiac rate and of systemic blood pressure; meanwhile, during QS, the newborn appears motionless and with regular breathing. A score of 1 was assigned if the TA was recognizable; a score of 2 if in an interval of 1 h it was possible to recognize a complete sleep cycle (two different states). If the indeterminate sleep pattern was present, a score of 0 was given (because it does not exist in the healthy newborn) [33,34].

- Seizures (S): If seizures were reported in clinical history, they were always annotated with the time of beginning and the duration of electrographic seizures and/or status epilepticus. We distinguished three conditions: status epilepticus (score: 0), electroencephalographic seizures (score: 1), and lack of seizures (score: 2). Status epilepticus is a clinical situation where an epileptic seizure (generalized or focal, motor or not) is prolonged for more than 20 min or the crises are repeated at very short intervals (less than a minute) such as to configure a continuous epileptic state (score: 0). An electrographic seizure (score: 1) was defined as a sudden, repetitive, stereotyped electrographic discharge of theta or delta rhythmic waves, alpha activity, or “sharp waves”, with a defined beginning, an intermediate evolution, and a precise end, lasting at least 10 s on almost one EEG channel. Suspected clinical seizures without an EEG discharge were not considered seizures. Video recording EEG was used to assess the possible correlation of clinical signs, if not reported in the clinical data available. For the absence of seizures, a score 2 was assigned [35].

TIMING
In our study, we analyzed EEGs at seven time intervals. A period of approximately 45 min was evaluated for each time interval. The first recording coincided with the first available EEG of the newborn, which was collected within the first 6 h of life (A). The second EEG assessment corresponded to 6 h after the first one (B = A + 6 h). The third EEG was performed 12 h after the second (C = B + 12 h), and the fourth and fifth were performed 24 h after the previous one (D = C + 24 h and E = D + 24 h). The sixth coincided with 6 h after the fifth (F = E + 6 h), and the last one was recorded on between the 9th and the 15th day of extrauterine life (G = 9–10 days after birth). If the last EEG available was recorded earlier, at 7–9 days old, it was considered only if it scored more than 9 points. Recordings earlier than the 7th day of life were not evaluated.
ANNOTATIONS AND SPECIAL CASES
  - If TA and bursts were not recognizable at that time interval, but a coherent background activity was present, a score of 1 point was assigned for each parameter characterizing the burst: BD, BA, and BM (total 3 points).
  - If continuous artifacts were present, the assessment was not possible (score = X/NA, not assessable)
  - Burst suppression (also called paroxysmal), which presents long periods of inactivity interrupted by bursts of abnormal activity of spikes, slow waves, or theta or beta rhythms shorter than <10 s, since it is very suggestive of HIE, was scored 1 point (1 point for all the six parameters).
  - Inactive EEG was scored 0.
